# SART-Type Image Reconstruction from a Limited Number of Projections with the Sparsity Constraint

**DOI:** 10.1155/2010/934847

**Published:** 2010-04-26

**Authors:** Hengyong Yu, Ge Wang

**Affiliations:** SBES Division and ICTAS Center for Biomedical Imaging, VT-WFU School of Biomedical Engineering and Sciences, Virginia Polytechnic Institute and State University, Blacksburg, VA 24061, USA

## Abstract

Based on the recent mathematical findings on solving the linear inverse problems with sparsity constraints by Daubechiesx et al., here we adapt a simultaneous algebraic reconstruction technique (SART) for image reconstruction from a limited number of projections subject to a sparsity constraint in terms of an invertible compression transform. The algorithm is implemented with an exemplary Haar wavelet transform and tested with a modified Shepp-Logan phantom. Our preliminary results demonstrate that the sparsity constraint helps effectively improve the quality of reconstructed images and reduce the number of necessary projections.

## 1. Introduction

Worldwide there are growing concerns on radiation induced genetic, cancerous, and other diseases [1–3]. Computed tomography (CT) is considered as a radiation-intensive procedure, yet it becomes more and more common. In the mid-1990s, CT scans only accounted for 4% of the total X-ray procedures but they contributed 40% of the collective dose [4]. The introduction of helical, multislice, and cone-beam technologies has increased and continues the increasing usage of CT [5, 6]. In US, the number of CT examinations has been estimated as high as nearly 60 million in 2002, which account for 15% of imaging procedures and 75% of the radiation exposure [4]. In June 2007, the *New York Times* reported that “*the per-capita dose of ionizing radiation from clinical imaging exams in the U.S. increased almost 600% from 1980 to 2006.*” More recently, in a high-profile article on the rapid growth in CT use and its associated radiation risks [3], Brenner and Hall estimated that “*on the basis of such risk estimates and data on CT use from 1991 through 1996, it was estimated that about 0.4*%* of all cancers in the United States may be attributable to the radiation from CT studies. By adjusting this estimate for current CT use, this estimate might now be in the range of 1.5 to 2.0*%.” Facing the increasing radiation risk, the well-known As Low As Reasonably Achievable (ALARA) principle is widely accepted in the medical community. One of the practical strategies is to reduce the number of necessary projection. 

Very interestingly, an elegant theory of compressive sampling or compressive sensing (CS) has recently emerged which shows that high-quality signals and images can be reconstructed from far fewer measurements than what is usually considered necessary according to the Nyquist sampling theorem [7, 8]. The main idea of CS is that most signals are sparse in an appropriate orthonormal system; that is, a majority of their coefficients are close or equal to zero, when represented in the proper domain. Typically, CS starts with taking a limited amount of samples in a much less correlated basis, and then the signal is exactly recovered with an overwhelming probability from the limited amount of data via the *ℓ*_1_ norm minimization. For example, the discrete gradient sparsifying transform has been widely utilized in CS-inspired CT reconstruction [9, 10], which was also referred to as the total variation minimization [11]. However, because the discrete gradient transform does not satisfy the restricted isometry property (RIP) required by the CS theory and is not invertible in general, such a reconstruction does not always convey the medically precise information. In particular, when a small number of projections are collected by a CT scanner, data noise may hide tumor-like structures in the TV-minimization-based reconstruction [12]. 

The above problem can be overcome using an invertible sparsifying transform such as a wavelet transform for image compression. For an object of interest such as a medical image, we can find an orthonormal basis (in a more general setting, a frame) to make the object sparse in terms of significant transform coefficients. Then, we can perform image reconstruction from a limited number of projections by minimizing the corresponding *ℓ*_1_ norm. Based on the recent mathematical findings made by Daubechies et al. [13, 14], here we will adapt a simultaneous algebraic reconstruction technique (SART) [15] for image reconstruction from a limited number of projections subject to a sparsity constraint in terms of an invertible sparsifying transform. 

This paper is organized as follows. In the next section, the mathematical principles will be summarized. In the third section, a SART-type reconstruction algorithm will be developed with a sparsity constraint. In the fourth section, preliminary numerical simulation results will be presented. In the last section, the related issues will be discussed. 

## 2. Mathematical Principles

Daubechies and her collaborators proposed a general iterative thresholding algorithm to solve linear inverse problems regularized by a sparsity constraint and proved its convergence [13, 14]. Their approach can be directly applied for the CT reconstruction from a limited number of projections. Their major results can be summarized as follows. 

Let **f** = [*f*_1_,*f*_2_,…,*f*_*N*_]^*T*^ ∈ ℝ^*N*^ be an object function and **g** = [*g*_1_,*g*_2_,…,*g*_*M*_]^*T*^ ∈ ℝ^*M*^ a set of measurements. Usually, they are linked by

(1) g=Af+e,

where **A** = (*a*_*mn*_) ∈ ℝ^*M*^ × ℝ^*N*^ is the linear measurement matrix, and **e** ∈ ℝ^*M*^ the measurement noise. Let us define the *ℓ*_*p*_ norm of the vector **g** as

(2) $${||g||}_{p}={\left(\overset{M}{\underset{m=1}{\sum }}g_{m}^{p}\right)}^{1/p}.$$

In practical applications, we usually omit the subscript *p* when *p* = 2. To estimate **f** from **g**, one can minimize the discrepancy Δ(**f**)

(3) $$\Delta \left(f\right)={||g-Af||}^{2}.$$

When system (1) is ill posed, the solution to (3) is not satisfactory, and additional constraints are required to regularize the solution. Particularly, given a complete basis (**φ**_*γ*_)_*γ*∈Γ_ of the space ℝ^*N*^ satisfying **f** = ∑_*γ*∈Γ_〈**f**, **φ**_*γ*_〉**φ**_*γ*_, and a sequence of strictly positive weights **w** = (*w*_*γ*_)_*γ*∈Γ_, we define the functional Φ_**w**,*p*_(**f**) by

(4) $$\Phi_{w,p}\left(f\right)=\Delta \left(f\right)+\underset{\gamma \in \Gamma }{\sum }2w_{\gamma }{\left|\langle f,\varphi_{\gamma }\rangle \right|}^{p},$$

where 〈·, ·〉 represents the inner product and 1 ≤ *p* ≤ 2. Using the *ℓ*_*p*_ norm definition (2), let us define the *ℓ*_*p*_ norm of a matrix operator **A** as

(5) $${||A||}_{p}=\underset{f\neq 0}{\max\;}\left(\frac{{||Af||}_{p}}{{||f||}_{p}}\right).$$

Let **A** ^*T*^ be the transpose matrix of **A**, the operator **A** in (1) is bounded, and ||**A** ^*T*^ **A**|| < *C*. In the following, we will assume *C* = 1 because **A** can always be renormalized. To find an estimate of **f** from **g** under the *ℓ*_*p*_ norm regularization term ∑_*γ*∈Γ_2*w*_*γ*_|〈**f**,**φ**_*γ*_〉|^*p*^, we can minimize Φ_**w**,*p*_(**f**) defined in (4). The minimizer of Φ_**w**,*p*_(**f**) can be recursively determined by the soft-thresholding algorithm:

(6) $$f^{k}={𝕊}_{w,p}\left(f^{k-1}+A^{T}\left(g-Af^{k-1}\right)\right),$$

where *k* = 1,2,… is the iteration number, **f** ^0^ the initial value in ℝ^*N*^, and

(7) $${𝕊}_{w,p}\left(f\right)=\underset{\gamma \in \Gamma }{\sum }S_{w_{\gamma },p}\left(\langle f,\varphi_{\gamma }\rangle \right)\varphi_{\gamma }$$

with *S*_*w*,*p*_ = (*F*_*w*,*p*_)^−1^ being a one-to-one map from ℝ to its self for *p* > 1 with

(8) $$F_{w,p}\left(x\right)=x+wp\mathrm{sgn}\;\left(x\right){\left|x\right|}^{p-1}.$$

Particularly,

(9) $$S_{w,3/2}\left(x\right)=\{\begin{array}{cc} x-\frac{3w\left(\sqrt{9w^{2}+16\left|x\right|}-3w\right)}{8} & \text{if}\;\;x\geq 0,\\ x+\frac{3w\left(\sqrt{9w^{2}+16\left|x\right|}-3w\right)}{8} & \text{if}\;\;x<0. \end{array}$$

When *p* = 1, we can set [13]

(10) $$S_{w,1}\left(x\right)=\{\begin{array}{cc} x-w & \text{if}\;\;x\geq w,\\ 0 & \text{if}\;\;\left|x\right|<w,\\ x+w & \text{if}\;\;x\leq -w. \end{array}$$

The main result of Daubechies et al. in [13] is that the solution of (6) is convergent. 

Unfortunately, the convergence speed of (6) is very slow. To facilitate practical applications, an accelerated projected gradient method was then developed [14]. When *w*_*γ*_ = *τ* for all *γ* ∈ Γ, Φ_**w**,*p*_(**f**) can be rewritten as

(11) $$\Phi_{w,p}\left(f\right)=\Phi_{\tau ,p}\left(f\right)=\Delta \left(f\right)+\underset{\gamma \in \Gamma }{\sum }2\tau {\left|\langle f,\varphi_{\gamma }\rangle \right|}^{p}.$$

Denote the minimizer of (11) as f* and define

(12) $$R\left(f^{*},p\right)={\left(\underset{\gamma \in \Gamma }{\sum }{\left|\langle f^{*},\varphi_{\gamma }\rangle \right|}^{p}\right)}^{1/p},$$

which is the *ℓ*_*p*_ norm radius of f* in the sparse space, we have the accelerated projected gradient algorithm

(13) $$f^{k}={\mathbb{P}}_{R(f^{*},p)}\left(f^{k-1}+\beta^{k}r^{k}\right),$$

where

(14) $$\begin{array}{c} r^{k}=A^{T}\left(g-Af^{k-1}\right),\;\;\beta^{k}={||r^{k}||}^{2}/{||Ar^{k}||}^{2},\\ {\mathbb{P}}_{R(f^{*},p)}\left(f\right)={𝕊}_{\mu ,p}\left(f\right)=\underset{\gamma \in \Gamma }{\sum }S_{\mu ,p}\left(\langle f,\varphi_{\gamma }\rangle \right)\varphi_{\gamma }, \end{array}$$

with an adapted soft-threshold *μ* = *μ*(*R*(f*, *p*), **f**) depending on *R*(f*, *p*) and **f**. When *R*(**f**, *p*) ≤ *R*(f*, *p*), *μ*(*R*(f*, *p*), **f**) = 0 and *ℙ*_*R*(f*,*p*)_(**f**) = **f**. When *R*(**f**, *p*) > *R*(f*, *p*), the adapted threshold *μ* should be chosen to satisfy

(15) $$R\left({\mathbb{P}}_{R(f^{*},p)}\left(f\right),p\right)=R\left({𝕊}_{\mu ,p}\left(f\right),p\right)=R\left(f^{*},p\right).$$

Regarding algorithm (13), we have several comments in order. First, although Daubechies et al. only proved the convergence for the case *p* = 1 [14], we believe that it should stand for 1 ≤ *p* ≤ 2. Second, while we have previously assumed that ||**A** ^*T*^ **A**|| < *C* and *C* = 1, it can be proved that the algorithm (13) holds for any positive *C*. Third, it is generally impossible to know the exact value of *R*(f*, *p*) but we can have an approximate estimate. 

## 3. Algorithm Development

**Figure 1:**
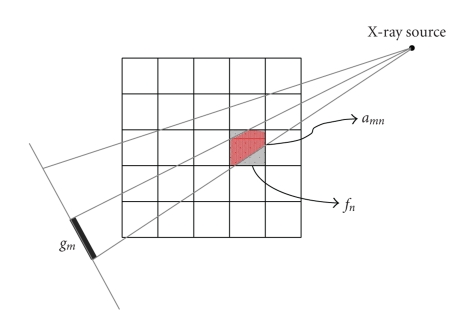
Projection model of a discrete image in fan-beam geometry.

In the context of image reconstruction, each component of the function **f** in (1) is interpreted as an image pixel with *N* being the total pixel number. Each component of the function **g** is a measured datum with *M* being the product of the number of projections and the number of detector elements. In fan-beam geometry with a discrete image grid, the *n*th pixel can be viewed as a rectangular region with a constant value *f*_*n*_, the *m*th measured datum *g*_*m*_ as an integral of areas of pixels partially covered by a narrow beam from an X-ray source to a detector element and respectively weighted by the corresponding X-ray linear attenuation coefficients. Thus, the component *a*_*mn*_ in (1) can be understood as the interaction area between the *n*th pixel and the *m*th fan-beam path (Figure 1). While the whole matrix **A** represents the forward projection, **A** ^*T*^ implements the back projection. The SART-type solution to (1) can be written as [15]

(16) $$f_{n}^{k}=f_{n}^{k-1}+\lambda^{k}\frac{1}{a_{+n}}\overset{M}{\underset{m=1}{\sum }}\frac{a_{mn}}{a_{m+}}\left(g_{m}-A_{m}f^{k-1}\right),$$

where *a*_+*n*_ = ∑_*m*=1_^*M*^ *a*_*mn*_ > 0, *a*_*m*+_ = ∑_*n*=1_^*N*^ *a*_*mn*_ > 0, **A**_*m*_ is the *m*th row of **A**, *k* the iteration index, and 0 < *λ* ^*k*^ < 2 a free relaxation parameter. Let Λ^+*N*^ ∈ ℝ^*N*^ × ℝ^*N*^ be a diagonal matrix with Λ_*nn*_^+*N*^ = 1/*a*_+*n*_ and let Λ^*M*+^ ∈ ℝ^*M*^ × ℝ^*M*^ be a diagonal matrix with Λ_*mm*_^*M*+^ = 1/*a*_*m*+_, then (16) can be rewritten as

(17) $$f^{k}=f^{k-1}+\lambda^{k}{\tilde{r}}^{k},$$

with

(18) $${\tilde{r}}^{k}=\Lambda^{+N}A^{T}\Lambda^{M+}\left(g-Af^{k-1}\right).$$

Due to the introduction of **Λ** ^+*N*^ and **Λ** ^*M*+^, (18) cannot be directly applied to solve (13). However, we can modify (18) to obtain a new **r** ^*k*^ defined as

(19) $$r^{k}=\frac{||A^{T}||}{||\Lambda^{+N}A^{T}\Lambda^{M+}||}{\tilde{r}}^{k}=\alpha {\tilde{r}}^{k}.$$

Substituting (19) into (13), we have a SART-type algorithm

(20) $$f^{k}={\mathbb{P}}_{R(f^{*},p)}\left(f^{k-1}+\alpha \beta^{k}{\tilde{r}}^{k}\right),$$

with *β* ^*k*^ = ${||{\tilde{r}}^{k}||}^{2}/{||A{\tilde{r}}^{k}||}^{2}$. The heuristic rationale for the above modification is to incorporate the SART-type weighting scheme for a more uniform convergence behavior. Now, our task is to estimate *α*. Since ||**A** ^*T*^|| = ||**A**||, we have

(21) $$\alpha^{2}=\frac{{||A^{T}||}^{2}}{{||\Lambda^{+N}A^{T}\Lambda^{M+}||}^{2}}=\frac{||A^{T}||\cdot ||A||}{||\Lambda^{+N}A^{T}\Lambda^{M+}||\cdot ||\Lambda^{M+}A\Lambda^{+N}||}.$$

Let **I** ∈ ℝ^*N*^ be the vector with all whose components being “1”. We have ||**A** ^*T*^ **A**||_1_ = ||**A** ^*T*^ **A**||_*∞*_ = max _1≤*n*≤*N*_(**A** ^*T*^ **A** **I**) because **A** ^*T*^ **A** is a symmetric matrix. Hence, we have

(22) $$||A^{T}A||\leq \sqrt{{||A^{T}A||}_{1}{||A^{T}A||}_{\infty }}=\underset{1\leq n\leq N}{\max\;}\left(A^{T}AI\right).$$

Similarly, we have

(23) $$\begin{array}{cc} & ||\Lambda^{+N}A^{T}\Lambda^{M+}\Lambda^{M+}A\Lambda^{+N}||\\ & \leq \sqrt{{||\Lambda^{+N}A^{T}\Lambda^{M+}\Lambda^{M+}A\Lambda^{+N}||}_{1}{||\Lambda^{+N}A^{T}\Lambda^{M+}\Lambda^{M+}A\Lambda^{+N}||}_{\infty }}\\ & =\underset{1\leq n\leq N}{\max\;}\left(\Lambda^{+N}A^{T}\Lambda^{M+}\Lambda^{M+}A\Lambda^{+N}I\right). \end{array}$$

That is,

(24) $$\alpha^{2}=\frac{{\max\;}_{1\leq n\leq N}\;\left(A^{T}AI\right)}{\max\;\left(||\Lambda^{+N}A^{T}\Lambda^{M+}\Lambda^{M+}A\Lambda^{+N}I||\right)}\alpha_{0}^{2},$$

with

(25) $$\alpha_{0}^{2}=\frac{{\max\;}_{1\leq n\leq N}\left(\Lambda^{+N}A^{T}\Lambda^{M+}\Lambda^{M+}A\Lambda^{+N}I\right)}{||\Lambda^{+N}A^{T}\Lambda^{M+}||\cdot ||\Lambda^{M+}A\Lambda^{+N}||}\frac{||A^{T}||\cdot ||A||}{{\max\;}_{1\leq n\leq N}\left(A^{T}AI\right)}.$$

In practical applications, we can set *α*_0_ to a reasonably large constant such as 2.0 in our simulation in the next section. If the algorithm does not converge, we can reduce *α*_0_ until the algorithm converges. 

For a basis (**φ**_*γ*_)_*γ*∈Γ_ of the space ℝ^*N*^, in which **f** has a sparse representation. Our SART-type CT algorithm regularized by sparsity can be summarized in the following pseudocode:

S1 Initialize *α*_0_, **f** ^(0)^ and *k*;
S2 Estimate *R*(f*, *p*);
S3 Precompute *α*, *a*_+*n*_ and *a*_*m*+_;
S4 Update the current estimation iteratively:
  S4.1 *k* : = *k* + 1;
  S4.2 ${\tilde{r}}^{k}:=\Lambda^{+N}A^{T}\Lambda^{M+}(g-Af^{k-1})$;
  S4.3 $\beta^{k}:={||{\tilde{r}}^{k}||}^{2}/{||A{\tilde{r}}^{k}||}^{2}$;
  S4.4 ${\tilde{f}}^{k}:=f^{k-1}+\alpha \beta^{k}{\tilde{r}}^{k}$;
  S4.5 Compute the sparse transform $\phi_{\gamma }:=\langle {\tilde{f}}^{k},\varphi_{\gamma }\rangle$ for *γ* ∈ Γ;
  S4.6 Estimate the adapted threshold *μ*;
  S4.7 Perform the soft-thresholding ${\tilde{\phi }}_{\gamma }:=S_{\mu ,p}\left(\phi_{\gamma }\right)$;
  S4.8 Perform the inverse sparse transform $f^{k}:=\sum_{\gamma \in \Gamma }{\tilde{\phi }}_{\gamma }\varphi_{\gamma }$;
S5 Go to S.4 until certain convergence criteria are satisfied.

In the above pseudocode, S.4.5 represents a sparse transform in a basis (**φ**_*γ*_)_*γ*∈Γ_. It can be either orthonormal (e.g., Fourier transform) or nonorthonormal, and *ϕ*_*γ*_ is the corresponding coefficient in the sparse space. In S.4.6, the adapted threshold *μ* can be estimated by a dichotomy searching method. S.4.7 performs the inverse sparse transform. Finally, the stopping criteria for S.5 can be either the maximum iteration number is reached or the relative reconstruction error (RRE) comes under a predefined threshold [14]:

(26) $$E^{k}=\frac{||f^{k}-f^{*}||}{||f^{*}||}\times 100.$$

## 4. Numerical Simulation

The above-proposed algorithm was implemented in MatLab. To demonstrate its validity, we performed several numerical tests assuming a circular scanning locus of radius 57.0 cm in fan-beam geometry. The object was a 128 × 128 modified Shepp-Logan phantom in a compact support with a radius of 10.0 cm. We used an equispatial virtual detector array of length 20.0 cm. The detector was centered at the system origin and made perpendicular to the direction from the system origin to the X-ray source. The detector array consisted of 128 elements. The well-known “Haar” wavelet transform was selected to derive a sparse representation. While the pixel number of the original phantom image was 16384, there were only 1708 nonzero wavelet coefficients. In our preliminary study, the *ℓ*_1_-norm was focused as suggested by the CS theory. For each of our selected numbers of projections over a full-scan range, we equiangularly acquired the corresponding projection dataset based on the discrete projection model as shown in Figure 1. The stopping criterion in S.5 was defined as either the maximum iteration number 20,000 was reached or the RRE became less than 0.1%. 

From each acquired dataset, we first reconstructed the image using the algorithm developed in Section 3, which is called “Scheme-A.” For comparison, we also run our codes without S.4.6–S.4.8. This strategy is an adapted SART-type reconstruction without regularization with the sparsity constraint, which is called “Scheme-B.” In reality, the real solution f* is usually unknown, Daubechies et al. suggested an interior algorithm that could slowly increase the radius of the solution in each iteration step [14]. Thus, we also modified our algorithm described in Section 3 by replacing *R*(f*, *p*) as *R* ^*k*^ = (0.4 + 0.6(*k*/20000)^0.05^)*R*(f*, *p*) in each iteration step *k* to implement the corresponding version of interior algorithm, which is called “Scheme-C.” 

**Figure 2:**
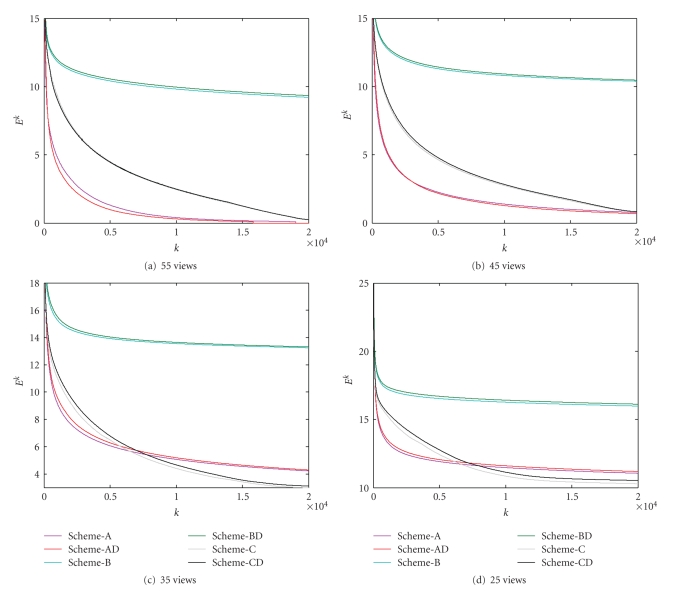
Relative error curves for various reconstructions from noise-free projections. The numbers of projections were set to (a) 55, (b) 45, (c) 35, and (d) 25, respectively, where the horizontal and vertical axes represent the iteration index *k* and the corresponding log error *E* ^*k*^, respectively.

**Figure 3:**
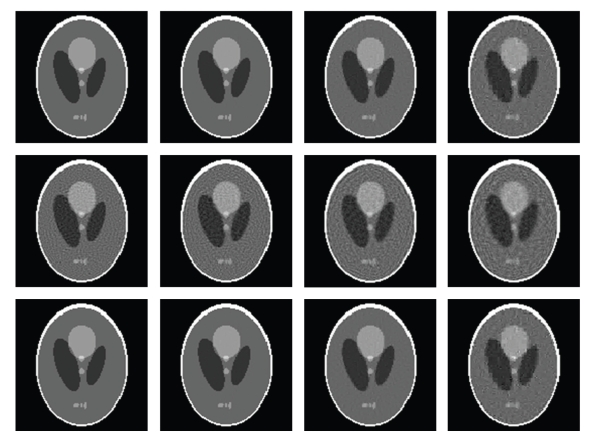
Reconstructed 128 × 128 images from noise-free projection datasets. The 1st, 2nd, and 3rd rows are reconstructed using Scheme-A, Scheme-B, and Scheme-C, respectively, and the 1st, 2nd, 3rd, and 4th columns are from 55, 45, 35, and 25 views respectively (the display window: [0  0.5]).

In the numerical simulation, after the stopping criteria were met, the iteration numbers and relative errors were listed in Table 1 with respect to different numbers of projections. The corresponding relative error convergence curves were plotted in Figure 2. The reconstructed images were shown in Figure 3. From the results in Table 1, Figures 2 and 3, we have several observations. When the number of projections was 55, Scheme-A reached a 0.1% RRE after 19040 iterations. Because 0.1% was really small, the corresponding reconstructed image would serve as a gold standard for all other reconstructed images. First of all, in any tested cases either Scheme-A or Scheme-C performed far better than Scheme-B, which confirmed that the sparse regularization did help improve the reconstructed image quality. Initially, the convergence speed of Scheme-A was faster than Scheme-C. However, after a number of iterations, the convergence speed of Scheme-A became slower than Scheme-C. If the ill posedness of the problem was not too bad, such as the cases of 55 and 45 projections, both Scheme-A and Scheme-C could perform well. When the problem was rather ill posed, such as the cases of 35 and 25 projections, Scheme-C would perform better than Scheme-A. 

Compared to the original algorithm proposed by Daubechies et al., one unique feature of the proposed SART-type algorithm is the weighting functions Λ^*M*+^, Λ^+*N*^ and the associated constant *α* for a more uniform converging behavior. To demonstrate this advantage, we modified our codes into a direct implementation of the algorithm described in [14] by forcing *α* = 1.0 and setting Λ^*M*+^ and Λ^+*N*^ to unit diagonal matrices. The aforementioned reconstruction strategies were named as “Scheme-AD,” “Scheme-BD,” and “Scheme-CD” and tested, respectively. The corresponding stopping conditions were listed in Table 2 with respect to the number of projections. The relative error curves were plotted in Figure 2. It can be observed in Figure 2 that when the problem was not too under-determined, such as in the cases of 55 and 45 projections, the proposed methods did not perform significantly better, and might do even worse (e.g., Scheme-AD was actually better than Scheme-A in the case of 55 projections). When the problem was seriously under-determined, such as in the cases of 35 and 25 projections, the proposed algorithms performed better than their direct implementation counterparts. 

**Figure 4:**
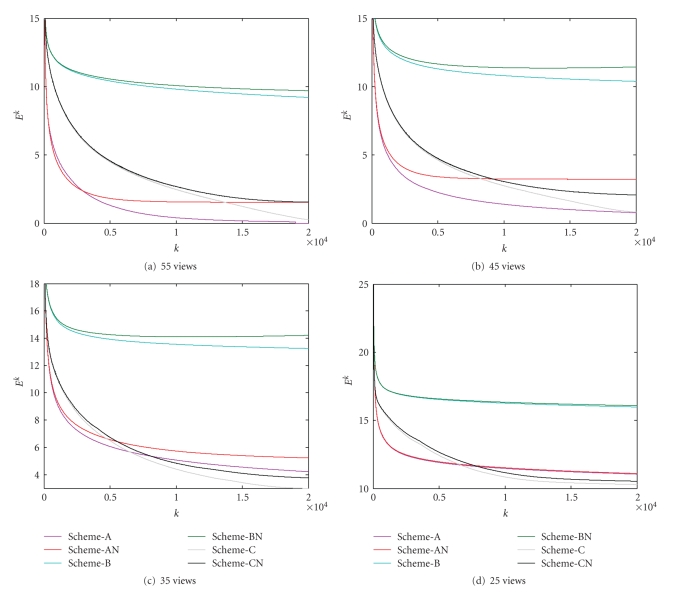
Relative error curves for various reconstructions from noisy projection. The numbers of projections were set to (a) 55, (b) 45, (c) 35, and (d) 25, respectively, where the horizontal and vertical axes represent the iteration index *k* and the corresponding log error *E* ^*k*^, respectively.

**Figure 5:**
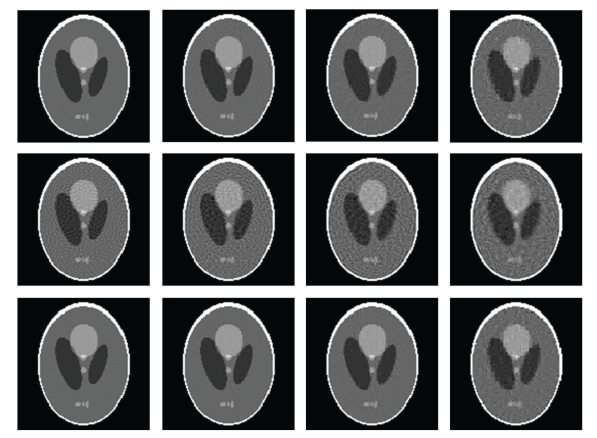
Counterpart of Figure 3 in the case of noisy projection datasets.

In practical applications, measurement noise is unavoidable. It is always important to use a stable algorithm for noisy data. To test the noise characteristic and stability of the proposed algorithms, we repeated the aforementioned reconstruction tests using “Scheme-A,” “Scheme-B,” and “Scheme-C” with projections bearing 0.1% Gaussian noise, which are denoted as “Scheme-AN,” “Scheme-BN,” and “Scheme-CN”, respectively. The corresponding stopping conditions were listed in Table 3 with respect to the number of projections. The converging curves were plotted in Figure 4. The reconstructed images were in Figure 5. It can be seen from the above results that the proposed algorithms produced similar relative error curves for noisy datasets compared to the noise-free counterparts. Due to noise in the projections, all the reconstructed images from noisy datasets generally had larger RREs than those from noise-free datasets given an iteration number. Note that Scheme-AN had a better performance than “Scheme-A” in the initial iterations in the case of 55 views. Our interpretation to this phenomenon is that in the initial iterations the discrepancy Δ(**f**) may be relatively large, which implies that Δ(**f**) may dominate the total cost functional Φ_**w**,*p*_(**f**), and the regularization effect of the *ℓ*_1_-norm would be small. 

## 5. Discussions and Conclusion

Although the above CS-based reconstruction algorithms have been accelerated relative to the previous benchmark [14], the convergence speed is still slow for large-scale images and/or very ill-posed conditions. In the future, we could use the state-of-the-art computing techniques to speedup the convergence, such as ordered-subset [15] and multiscale computing [16] techniques. At the same time, we should optimize the reconstruction parameters and imaging protocols as well. 

For the modified Shepp-Logan phantom, the *ℓ*_1_-norm seems giving the best performance than any other *ℓ*_*p*_ norm with 1 < *p* ≤ 2 in a Haar space. However, it does not imply that the *ℓ*_1_-norm is the best option for any application. In fact, our algorithm was implemented for any 1 ≤ *p* ≤ 2. For a specific application, the optimal *p* may be studied. 

Furthermore, this orthonormality of the wavelet transform used in this study is not necessary. If an image can be sparsely expanded in a certain basis or frame, the *ℓ*_*p*_-norm minimization can be in principle performed to regularize the reconstruction process. Since there exist many compression methods for medical images, we should evaluate representative bases and frames for sparse representations and CS-based reconstruction methods. The heuristic rule is to achieve a minimal compression ratio. 

In conclusion, we have developed a SART-type reconstruction algorithm based on the recent mathematical findings by Daubechies et al. Our preliminary simulation results have confirmed its merits and suggested research directions. Because the approach accommodates any 1 ≤ *p* ≤ 2 and any sparse expansion, there should be a large room for further improvements of the algorithm performance. 

## Figures and Tables

**Table 1:** Maximum iteration numbers and relative errors associated with each of three representative reconstruction schemes for different numbers of noise-free projections.

View number	Scheme-A	Scheme-B	Scheme-C
55	19040 (0.1000)	20000 (9.2080)	20000 (0.2734)
45	20000 (0.7689)	20000 (10.3855)	20000 (0.8261)
35	20000 (4.2200)	20000 (13.2479)	20000 (2.9895)
25	20000 (11.0556)	20000 (15.9855)	20000 (10.2940)

**Table 2:** Same as Table 1 but for the direct implementation counterparts of the proposed algorithms.

View number	Scheme-AD	Scheme-BD	Scheme-CD
55	15805 (0.1000)	20000 (9.3391)	20000 (0.2477)
45	20000 (0.6837)	20000 (10.4658)	20000 (0.8357)
35	20000 (4.2946)	20000 (13.3163)	20000 (3.1190)
25	20000 (11.1846)	20000 (16.1218)	20000 (10.5271)

**Table 3:** Same as Table 1 but for projection data bearing 0.1% Gaussian noise.

View number	Scheme-AD	Scheme-BD	Scheme-CD
55	20000 (1.5386)	20000 (9.7042)	20000 (1.5496)
45	20000 (3.2240)	20000 (11.4394)	20000 (2.0746)
35	20000 (5.2298)	20000 (14.2091)	20000 (3.7667)
25	20000 (11.0959)	20000 (16.0837)	20000 (10.5335)
